# Seasonal patterns and processes of migration in a long-distance migratory bird: energy or time minimization?

**DOI:** 10.1098/rspb.2024.0624

**Published:** 2024-06-05

**Authors:** Anders Hedenström, Linus Hedh

**Affiliations:** Department of Biology, Lund University, Ecology Building, 223 62 Lund, Sweden

**Keywords:** accelerometery, bird migration, flight, migration strategy, stopover, time minimization

## Abstract

Optimal migration theory prescribes adaptive strategies of energy, time or mortality minimization. To test alternative hypotheses of energy- and time-minimization migration we used multisensory data loggers that record time-resolved flight activity and light for positioning by geolocation in a long-distance migratory shorebird, the little ringed plover, *Charadrius dubius*. We could reject the hypothesis of energy minimization based on a relationship between stopover duration and subsequent flight time as predicted for a time minimizer. We found seasonally diverging slopes between stopover and flight durations in relation to the progress (time) of migration, which follows a time-minimizing policy if resource gradients along the migration route increase in autumn and decrease in spring. Total flight duration did not differ significantly between autumn and spring migration, although spring migration was 6% shorter. Overall duration of autumn migration was longer than that in spring, mainly owing to a mid-migration stop in most birds, when they likely initiated moult. Overall migration speed was significantly different between autumn and spring. Migratory flights often occurred as runs of two to seven nocturnal flights on adjacent days, which may be countering a time-minimization strategy. Other factors may influence a preference for nocturnal migration, such as avoiding flight in turbulent conditions, heat stress and diurnal predators.

## 1. Introduction

Migrating birds alternate periods of stopover, when energy is accumulated, and flight, when they exchange their stored energy for distance towards their goal. How birds execute their migration varies a lot, from the extreme of making a single non-stop flight, such as in the bar-tailed godwit, *Limosa limosa baueri,* flying from Alaska across the Pacific to New Zealand in the autumn [1], to a stepwise migration involving short flights between consecutive stopovers, as in some small shorebirds and many terrestrial birds [e.g. 2–5]. Where on this spectrum a species or specific population places itself is likely determined by the distribution of suitable stopover habitats along the route and whether major ecological barriers, such as seas and deserts, must be traversed. The flight step length(s) likely also depend(s) on migration strategy, i.e. whether mortality, energy or time minimization of migration is the prime selective driver.

Optimal migration theory (OMT) provides a framework from which adaptive (optimal) behaviours can be derived based on alternative optimization criteria [6–8]. Behaviours affected by alternative strategies include optimal stopover duration (t*) and the associated fuel load (f*) at departure, as well as optimal flight speeds [6,9]. The trade-off between staying or departing on a migratory flight depends on the diminishing utility in flight range of added fuel load, which in turn is contingent on the aerodynamic costs of carrying fuel [7], the experienced rate of fuel (energy) deposition, and search/settling energy and time costs [6]. Depending on how migrants respond to variation in these factors and a set of rules about how to evaluate information regarding variation in fuelling rate obtained along the migration route, alternative strategies of energy- and time-minimization migration can be tested.

Previous empirical tests of optimal stopover time and associated departure fuel loads have involved experimental manipulation of food availability at stopovers. In general, such experiments have found support for a time-minimization policy, with a few exceptions in support of energy minimization [10,11]. However, most studies refer to passerines, while critical tests of alternative migration strategies for other avian taxa are rare. Shorebirds are often considered representatives of the long-haul flight strategy with non-stop flights between a few discrete stopover sites [12–15]. This certainly applies to several species where prolonged non-stop flights have been demonstrated by satellite telemetry or positional loggers [15–17], with non-stop flights of between 7500 and 11 700 km that correspond to a flight duration of 7–8 days. To distinguish between alternative optimization strategies, it is advantageous to focus on a long-distance migrant that is not dependent only on a few possible stopovers, but that could migrate with shorter flights along a route with abundantly distributed potential stopovers. A candidate shorebird fulfilling these requirements is the little ringed plover, *Charadrius dubius* (henceforth LRP), breeding in Europe [18], which we chose as a study system. The presentation requires the introduction of some formalism, and a list of symbols and their definitions is found in table 1.

**Table 1.** List of symbols and abbreviations and their definitions.

symbol	definition
*c*	composite factor of range equation (km)
f, f*	fuel load, optimal fuel load
*f*_0_	search/settling energy cost
*f*_E_	optimal fuel load for energy minimization
*k*	daily fuel deposition rate as proportion of lean mass
*S*_exp_	expected instantaneous migration speed
*S*_inst_	instantaneous migration speed
*t*, t*	stopover time, optimal stopover time (days)
*Y*	flight range as a function of fuel load (km)
LRP	little ringed plover
MDL	multisensor data logger
OMT	optimal migration theory

The derivation of optimal fuel load, stopover duration and associated flight step depends on the flight range equation

1.1 $$Y=c\left(1-\frac{1}{\sqrt{1+f}}\right),$$

where *Y* is flight distance, *f* is the fuel load required to cover distance *Y*, and *c* is a composite factor including morphology, energy content of the fuel and energy conversion efficiency [6,7]. By substituting *f* with *kt*, where *k* is the daily fuel deposition rate expressed as a proportion of lean mass, the optimal fuel load, stopover duration and flight distance can be derived for search/settling energy and time costs, *f*_0_ and *t*_0_, upon arrival at new stopovers [19]. By differentiating equation (1.1), with *f* substituted with *kt*, with respect to time (*t*) we get an expression for the instantaneous migration speed as

1.2 $$S_{\mathrm{inst}}=\;\frac{dY}{dt}=\frac{c}{2}k{\left(1-\frac{Y}{c}\right)}^{3}.$$

**Figure 1.**
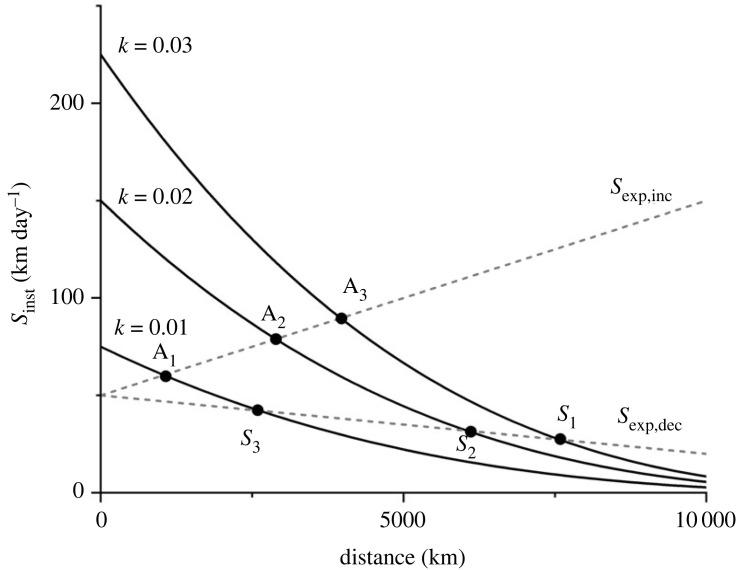
Optimal flight ranges (and associated fuel loads) in relation to instantaneous speed of migration (*S*_inst_) and expected migration speed (*S*_exp_) for time-minimizing migrants where fuel load is related to range according to equation (1.1). Solid lines represent different fuel deposition rates (*k*) and hatched lines represent alternative expected migration speeds along the route as increasing (*S*_exp,inc_) or decreasing (*S*_exp,dec_). In the case of *S*_exp,inc_ with low *k* (= 0.01) at the site of departure the optimal fuel load from site 1 is associated with range at A_1_, with successively increasing fuel loads at subsequent stopovers (A_2_, A_3_) as *k* increases along the route to medium (= 0.02) and high (= 0.03) rates. When expected migration speed decreases (*S*_exp,dec_) and fuelling rate is relatively high at the site of departure, it is optimal to exploit the first site longer and depart with a high fuel load (*S*_1_), with decreasing departure fuel loads at subsequent stopovers (*S*_2_, *S*_3_) when the rates drop. The figure is intended to show qualitatively how departure fuel loads are expected to vary under different gradients of food availability at stopovers along a migration route, but the quantitative optimal departure fuel loads are contingent on experienced fuelling rates and other external ecological factors.

In analogy with the marginal value theorem of foraging, a migrant should leave the current stopover when *S*_inst_ drops to the expected migration speed along the route, *S*_exp_. By using equation (1.2) the expected fuel loads, stopover duration and associated flight distance can be derived for situations of changing migration speed along the route (figure 1; cf. [20]). Based on these relationships, we formulated the following testable hypotheses:

(H1) If following an energy-minimization strategy, birds should depart from a stopover when reaching the same fuel load, $f_{E}^{*}$, which only depends on the search/settling energy cost, *f*_0_ [19]. If the fuel deposition rate (*k*) varies among stopovers, stopover duration will vary accordingly, but since the fuel load at departure is (near) constant the following flight duration will be constant, irrespective of the preceding stopover duration.
(H2) If birds follow a time-minimization policy with a fixed expectation of fuel deposition rate, *k*, along the route, then stopover duration and associated flight distance will vary as a response to local variation in *k*, and associated departure fuel load and flight distance will covary [6,19].
(H3) A migrant should leave the current stopover when the marginal rate of the instantaneous migration speed has dropped to the expected migration speed along the route (equation (1.2)). Depending on the expected migration speed along the route, a migrant will adjust stopover duration so that if *S*_exp_ is increasing, stopovers will be short, fuel loads relatively small and following flight steps short at the beginning of migration, but will increase successively along the route (figure 1). Contrary, if *S*_exp_ is decreasing along the route, stopovers will be relatively long, fuel loads large and flight steps long at the beginning of migration and will successively decrease with the progress of migration (figure 1). These situations are expected to apply to autumn and spring migration, respectively, where migration speed increases during autumn migration [21–23], while spring migration is against a resource gradient, with diminishing food abundance from the wintering to breeding area [14,24–27].
(H4) It has been suggested that the overall migration strategy may differ between spring and autumn, as indicated by faster flight speeds in spring than in autumn [28,29], since time-minimizing birds should fly at a faster airspeed than if minimizing cost of transport [9]. A compilation of seasonal migration and flight speeds showed that birds tended to migrate and fly faster in spring than autumn [30], although this is not evidence of seasonally differing strategies since the environment may provide seasonally differing conditions such as winds and food availability [31,32]. Anyway, if spring migration is time-selected and autumn migration is energy-selected we predict that flight speeds should be faster in spring than in autumn.

To evaluate alternative rules of energy- and time-minimization migration, we deployed multisensor data loggers (MDLs) to record flight routes, stopover durations and flight step lengths for LRPs breeding in southern Sweden. The MDLs record flight activity with high temporal resolution, which allows us to determine duration of stopovers and migratory flights of both autumn and spring migration. We can thereby address key predictions of OMT with respect to energy- and time-minimization strategies. It should be noted that, for birds using external energy while flying, such as cross-country thermal soaring or dynamic soaring, the relationship between fuel load and flight range differs from that of equation (1.1) [33], and consequently predictions about fuelling and flight strategies differ from those tested in the present paper.

## 2. Methods

### (a) Study population and trapping

We captured LRPs breeding at locations in the province of Skåne, south Sweden, where they breed in man-made water treatment ponds, gravel pits or other freshwater habitats. Adult birds were trapped in the nest using a circular walk-in trap with one funnel-shaped entrance, or a small spring trap, in the years 2016–2022. The birds were ringed using numbered aluminium rings and a combination of colour rings to allow field identification of individuals that potentially carry data loggers (see below) from a previous year's deployment. When handling the birds, we measured standard morphometrics, including wing, bill, head + bill, tail and tarsus lengths, and body mass using an electronic balance. Birds were sexed based on plumage characteristics, where females have more brown feathers in the otherwise black head marks and breast band, and a narrower and paler yellow eye orbital ring than males. Sexing was often facilitated by direct comparison of the members of a pair. In total, we deployed multisensor data loggers (MDLs) on 95 birds, including those that were deployed twice, and up to and including the 2022 season 27 birds were recaptured, corresponding to a 28% recapture rate.

### (b) M ultisensor data loggers

The multisensor data logger (MDL) comprises a real-time clock microcontroller, memory, an accelerometer, and temperature, pressure and light sensors. The accelerometer measures acceleration in three dimensions at a ±4*g* range at a sampling rate of 100 Hz. Only the vertical axis (*Z*) acceleration was used for activity analysis. Details about the hardware configuration and sampling routines can be found in [2]. For this study we do not use pressure or temperature data. The mass of the data logger was ≤1.25 g and on deployment the average body mass of the birds was 43.1 g, meaning the logger mass was 2.8% of the body mass on average.

### (c) Geolocation by light

The MDLs were pre-programmed with a calendar defining when to run the light-level measurements for position estimates. This approach is different from conventional geolocators in that our loggers only measured sequences of daily light cycles for a limited number of consecutive days. The reduction of measurement periods of light level substantially reduces the amount of data to be stored and prolongs operation time by minimizing the power consumption. In our study, we ran six measurement periods each of 5 days distributed over 1 year, starting on 15 July, 1 September, 1 November, 1 February, 15 April and 5 May. The timing of measurement sequences was selected to match periods of residency previously identified by conventional geolocators [18]. In this study, we adopt a Northern Hemisphere perspective, where spring migration is synonymous with pre-breeding migration and autumn migration equals post-breeding migration towards non-breeding areas.

We used the R software *GeoLight* for all steps in the analyses of light-level data [34]. To annotate twilight events we used the *twilightCalc*() function and a threshold value of 2 lux. Each twilight event was confirmed visually before proceeding. Positions were translated from light measurements into geographical positions using the *coord*() function. Because light intensity was only measured during a few short periods, conventional calibrations were not possible. Consequently, we initially set Sun elevation angles to −6°, i.e. making a civil twilight calibration. For seven loggers we adjusted the Sun elevation angles in 0.5° steps to obtain best calibration at 5.5° (*n* = 3), 5° (*n* = 2) and 4° (*n* = 2), respectively.

Adjustments were done by visually inspecting the discrepancy in latitude estimates between the two measurement periods in November and February if activity data indicated no movement, and for the positions derived in May, when we knew that the birds had returned to the breeding sites, adjustments were made to yield positions corresponding to south Sweden. This approach gives a somewhat less certain estimate of wintering positions compared with geolocators recording light continuously, but we obtained reasonable estimates of wintering areas, and major stopovers provided limited data.

Total migration distances were estimated as the sum of great circle distances between well-defined consecutive stopovers and the breeding/wintering sites. To define stopovers, we used light data during periods of residency as revealed by the activity data (see below). Especially in the autumn, birds tended to make longer detours, which resulted in somewhat longer migration distances in autumn than in spring, when migration route is more direct [18].

### (d) Flight activity

**Figure 2.**
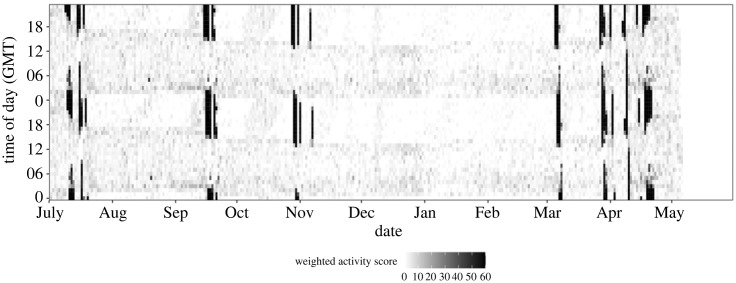
Migration actogram for a male little-ringed plover migrating between Sweden and south India in the season 2019–2020. To visualize behavioural rhythms an actogram shows data for two consecutive 24 h periods aligned vertically (*y*-axis) along the time *x*-axis. Periods of migratory flights are seen as black vertical bars, with medium and low activity indicated by shades of grey. Most flights were initiated in the evening and were performed during night-time, with the exception of occasional longer flights that extended into daytime. This individual (male) started migration on 10 July 2019 and reached its wintering location in southeast India (Andhra Pradesh) on 7 November, having flown for 150 h. Spring migration was initiated on 6 March 2020 and the bird arrived at the breeding site on 21 April. The total flight time was 148 h in the spring. The daily activity period is longer in August–September compared with December–January, which reflects the change in daylength with latitude and time. The active period is also time-shifted between summer and winter, which reflects the large longitudinal displacement of this individual.

Acceleration in the *Z*-axis was sampled every 5 min with runs of 10 measurements at five time points, each separated by 5 s. Each measurement is a sample during 100 ms at 100 Hz in the range ±4*g*. For each run, the mean of the values was subtracted from each of the 10 measurements to compensate for static gravity, and the recorded acceleration was considered as indicative of flight if at least 3 of the 10 values were greater than |*g*/3|, where *g* is acceleration due to gravity. Each 5 min sample was assigned the number of runs that indicate flight behaviour, i.e. (0, … , 5). Every hour a summary of results from all 12 runs was stored according to the distribution of the samples across the different activity categories (0, … , 5). If the bird is perched and motionless the data stored will be (12, 0, 0, 0, 0, 0), and if it is flying with continuous wing beats the data are (0, 0, 0, 0, 0, 12). To illustrate flight activity in graphical actograms the hourly recordings are coded as ‘black' to represent continuous flapping flight (0, 0, 0, 0, 0, 12), and ‘white' to represent no flight (12, 0, 0, 0, 0, 0), with shades of grey representing intermediate levels of activity (figure 2; [2,35]). The hourly activity data are shown in a double-plotted format for two consecutive days, where the data for the two days occur twice but shifted, which is a standard way to display activity data in chronobiology (figure 2).

### (e) Analysis of activity data

To identify flight periods based on the accelerometer data we derived weighted hourly activity scores by calculating the sum of each score multiplied by the number of events within each hour. Thus, the lowest score becomes 0 (0 × 12) and the highest possible score is 60 (5 × 12). We then identified all hours with a weighted score ≥50, which we defined as an activity level corresponding to flapping flight. In almost all cases these hours were associated with sequences of high scores that could be identified as periods of flight. Start and end times of a flight were defined around the above-defined flight period by subtracting all measurements (5 min periods) falling under a score of 3. Specifically, for a flight to be defined as ended, zero-scores must be present during that hour. Thus, the start and end points of flight periods, and hence flight duration, were calculated to a 5 min resolution, provided that all scores <3 and all scores ≥3 were recorded in sequence, respectively. Periods between well-defined flight periods were recorded as stopovers or winter/breeding site residency, depending on the season. In some cases, particularly at the end of flight periods, hourly weighted scores were below 50. In such cases, we examined the distribution of the raw activity scores (0–5) and defined the hour as *in flight* as long as no zero scores were recorded. If a zero score was recorded, we looked at the next hour to assess if the bird had landed by summing the number of zeros between the two hours. If that sum was ≥12 (corresponding to ≥1 h) the bird was considered to have landed; if it was not, then the bird had continued, and we considered the full sequence as a continuous flight. However, when calculating the duration of the flight all 5 min scores <3 were omitted. Regardless, there are several ecological reasons why these brief periods of apparent inactivity, which include lower weighted scores, should still be defined as *in flight*. First, lower scores could be due to descending flights, which may occur during mid-flight owing to altitude adjustments (e.g. [36]). Second, upon arrival at a location of residency (stopover or winter/breeding site) it is not uncommon for shorebirds to make shorter local movements. Third, even if a short landing (less than 1 h) occurs in mid-flight, we treat the prior and subsequent flights as continuous flights, because stops lasting less than 1 h are likely too short to allow refuelling [19,37]. If subsampling had partly or fully failed (e.g. if only 10 out of 12 or no scores were available during an hour) during a flight period, these samples were defined as zeros and were not included to calculate flight duration.

### (f) Data analysis

Migration starts with a period of fuelling in order to allow the first migratory flight [38]. However, in most cases the duration of this period is unknown since the bird may still be at the breeding or wintering site. One alternative could be to use published information about fuelling rates and estimate the time required to fuel for the first flight(s) [39]. However, to avoid the introduction of assumptions we discarded the first flights of a season when analysing the relationship between stopover duration and subsequent flight duration (see [40]). This approach is also not ideal since in cases when significant fuelling occurs before the first flight this fuel may also affect flights after the first flight if flights occur back-to-back, which in turn will weaken the true relationship between stopover duration and subsequent flight duration. Migratory flights often occurred as runs of two or more flights back-to-back with short intermediate stops during the daytime (figure 2). It is unlikely that significant fuel accumulation takes place on such short stops, whereas it is often the case that birds tend to lose some weight initially at new stopovers [37,41,42]. Therefore, we combined runs of back-to-back migratory flights with inter-flight stops of less than 0.6 day and analysed them as if energetically equivalent to non-stop flights of the same duration. We thereby assume that the stopover–flight dynamics should be stronger if using such combined flight duration instead of the actual (shorter) flight episodes as recorded. The criterion of combining flights only if stops are <0.6 day (about 14 h) ensures that birds that migrated during the night did at most stay the following day before resuming migration the next night.

### (g) Statistics and hypothesis testing

Statistical analyses were made using JMP^©^ Pro 16.0 (SAS Institute). We performed linear mixed models for the dependent variable with respect to the fixed factors of total migration distance, season, date, and interaction terms when appropriate. Because we had repeated measurements of a few individuals we included individual identity as a random factor in statistical analyses. For statistical models, date was normalized within the season so that day 1 is equal to the Julian date when the first individual commenced migration. In cases where the data did not meet the requirement of normally distributed residuals, we log_10_-transformed the data before analyses to obtain data that met the requirements for linear mixed models.

To evaluate our hypotheses (H1–H4) the following criteria will be used. We will refute H1 (see Introduction) if flight durations vary in relation to stopover duration but accept it if flight durations are relatively constant. Conversely, we will refute H2 if flight durations are relatively constant because of invariant departure fuel loads but accept it if stopover durations and the following flight durations covary. If resource gradients exist as assumed for autumn and spring apply for the LRP (H3), this will manifest as positive (autumn) and negative (spring) slopes, respectively, for flight step lengths (measured as flight duration) in relation to the advancement of the season. We accept this hypothesis if predicted relations are significant.

We test if the LRP shows seasonally differing flight speeds (H4) based on the seasonal total flight time between autumn and spring. If flight speed is higher in spring than in autumn we accept the hypothesis that spring migration is time-selected and autumn migration is energy-selected, while if flight speeds do not differ between seasons we reject the hypothesis.

The research was approved by the Malmö-Lund animal ethics committee (M33/13; M72/15).

## 3. Results

Out of 27 birds recaptured that carried MDLs we obtained data from 20 devices, representing 20 autumn migrations and 19 spring migrations. Most of the MDLs that did not contain data were recovered in the 2018 season, when for an unknown reason all devices failed to collect data.

### (a) Migration routes and distances

Wintering sites ranged across the sub-Saharan African continent from Senegal to East Africa (same individual two winters in Ethiopia/Somalia), with several individuals clustering in the Sahel region in the countries Mali, Niger, Nigeria and Chad (electronic supplementary material, figure S1). In autumn the total migration distance was on average 5974 km (range 4626–8287 km, *N* = 20) and in spring the mean was 5612 km (range 4161–7826 km, *N* = 19; *F*_1,23.2_ = 7.23, *p* = 0.0131).

### (b) Durations of flights

**Figure 3.**
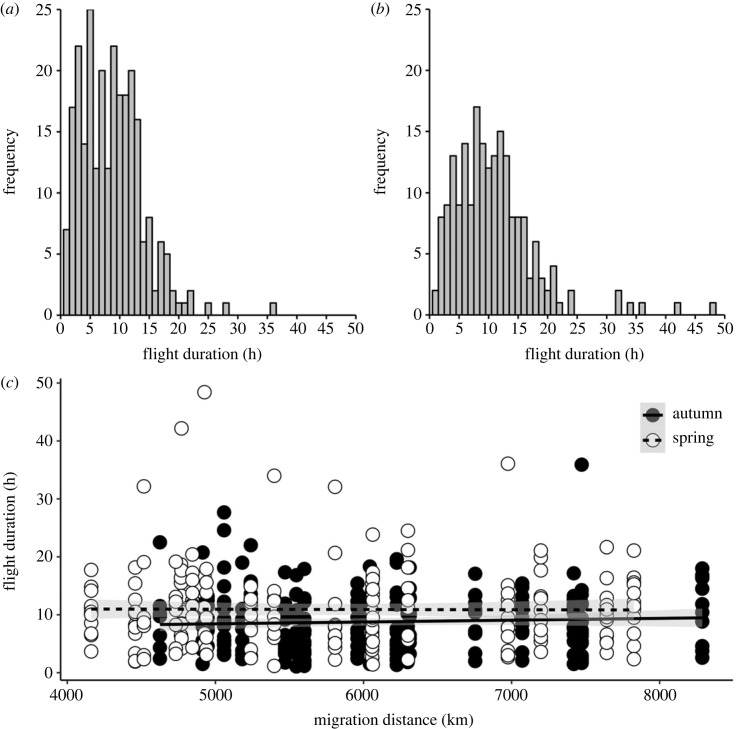
Frequency distribution of individual flight durations in little ringed plovers breeding in southern Sweden for (*a*) autumn migration and (*b*) spring migration. Panel (*c*) shows the durations of individual flights in relation to total migration distance for autumn (filled circles) and spring (open circles) migration. The lines indicate the linear regressions for autumn and spring migration, respectively. Shaded areas show the 95% confidence bands.

Mean duration of single flights in autumn was 8.8 h, but longer flights occurred occasionally with a maximum non-stop flight of 36 h (figure 3*a*). The distribution of flight durations was similar in spring with a tendency for fewer but longer flights (figure 3*b*), with an average of 10.9 h and the longest non-stop flight clocking 48 h. There was a significant effect of season on single flight durations (figure 3*c*; *p* = 0.0002; electronic supplementary material, table S1 for statistical details), with single flights being longer in spring than in autumn, but there was no significant effect of total migration distance (figure 3*c*; *p* = 0.80; electronic supplementary material, table S1) or the interaction between season and migration distance on flight durations (*p* = 0.54; electronic supplementary material, table S1).

**Figure 4.**
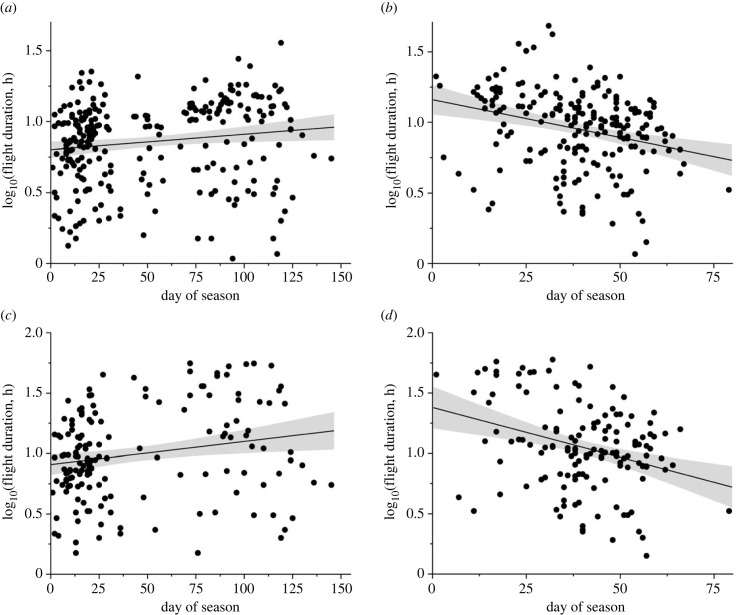
Migratory flight duration in relation to date within season during autumn (*a,c*) and spring (*b,d*). For (*a*) and (*b*) all data are shown as recorded by the MDLs, while in (*c*) and (*d*) flights separated by less than 0.6 day interruptions are combined. The relationships of all panels are statistically significant (see electronic supplementary material, table S3 for statistical details).

If the first flight of a season was excluded, there was significant relationships between flight durations and prior stopover durations for both single flights and combined runs of back-to-back flights (*p* < 0.0001; electronic supplementary material, table S2 and figure S2). Therefore, to test the hypothesis H3 we focus on the flight durations, which are not affected by any assumptions about fuel deposition rates before the first flight of a season. The single flight durations increased with the progress of the season in autumn (figure 4*a*), while in the spring the flight durations decreased with the progress of the season (figure 4*b*), with a significant interaction between day within season and season (*p* < 0.0001; electronic supplementary material, table S3a for statistics). When analysing combined back-to-back flights the same pattern was maintained as for the raw flight data (figure 4*c*,*d*; electronic supplementary material, table S3b for statistics). First migratory flights of a season were longer in spring (mean 16.8 h, *N* = 19) than in autumn (6.7 h, *N* = 20; *F*_1,23.2_ = 25.3, *p* < 0.0001).

### (c) Duration of migration, speed of migration and flight speed

The duration of autumn migration, from departure from the breeding site until arrival at the wintering site, was on average 90 days (range 19–123 days), while the corresponding duration of spring migration was 37 days (range 13–56 days). The difference was significant (*p* < 0.0001; electronic supplementary material, table S4). However, during autumn migration most birds (*N* = 18) made a relatively long stop (average 54 days, maximum 83 days), which is likely longer than the time required to fuel for the upcoming flight(s).

**Figure 5.**
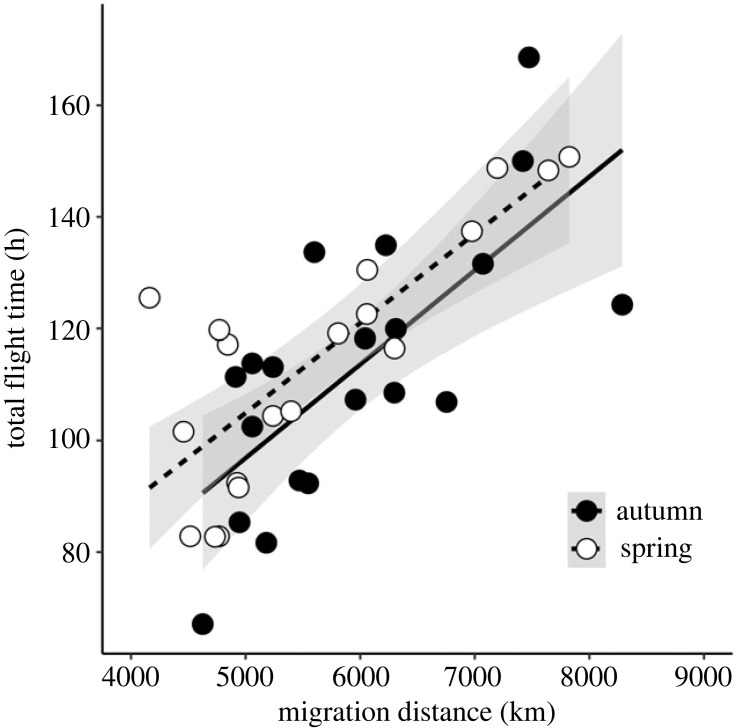
Total seasonal flight duration in relation to migration distance (great circle, km) for little ringed plovers breeding in southern Sweden. Filled circles represent autumn migration and open circles represent spring migration. For individual migration routes see electronic supplementary material figure S1. See electronic supplementary material, table S6 for statistical details.

The overall speed of migration was significantly slower in autumn than in spring (autumn mean = 76.7 km day^−1^, spring mean 168 km day^−1^; *p* = 0.0002; electronic supplementary material, table S5). However, these estimates do not account for the time required to fuel for the first flight(s) of a season [38], but they include the extended stop during autumn migration that most individuals made. As expected, seasonal total flight duration was strongly related to total migration distance (figure 5; *p* < 0.0001; electronic supplementary material, table S6), while season had no significant effect on total flight duration (*p* = 0.149; electronic supplementary material, table S6). Even if migration distances differed somewhat between seasons, there was no significant difference between seasons in estimated flight speed (*p* = 0.0619; electronic supplementary material, table S7). The estimated flight speed was 14.3 m s^−1^ (electronic supplementary material, table S7).

## 4. Discussion

### (a) Energy or time minimization

By using novel technology, we obtained detailed data on the migration process for a small shorebird, the little-ringed plover, that provided an unprecedented opportunity to evaluate which of alternative optimal migration strategies little-ringed plovers are using. We formulated four hypotheses (H1–H4) with testable predictions about stopover and flight step dynamics. Because both stopover durations and flight steps varied substantially, we reject the hypothesis that the birds follow a pure energy-minimization strategy (H1). Consequently, a related hypothesis that birds may follow a rule of thumb to always stop a fixed time can also be rejected (cf. [43]). Instead, there was a significant relationship between the duration of stopovers and subsequent flights, which suggests the birds follow a time-minimizing policy (H2), where birds are expected to adjust fuel loads and associated flight distances to experienced variation in fuel deposition rates [6,8,19]. Most previous studies testing between energy and time minimization refer to experimental manipulation of food at stopovers in passerines [10,11], and so this study not only represents a taxonomic extension of the use of time-minimization migration but also provides evidence based on natural flight/stopover behaviours over entire migration seasons. We also tested a corollary of the time-minimization hypothesis, which predicts how fuel loads and flight steps should vary when migration speed is expected to increase or decrease along the route (H3). Based on assumed, although highly likely, gradients of resources at stopovers for autumn (increasing) and spring (decreasing) migration we expect that correlation between flight step lengths (measured as duration) in relation to the progression of the season should be positive in autumn and negative in spring, which is what our data showed (H3 accepted). The observed diverging responses of stopover and flight dynamics with the progression of migration between the seasons therefore provide compelling support for the notion that a time-minimization strategy is adopted in both seasons. In a comparative study, it appeared that most bird species migrated faster in spring than in autumn [30], which again was considered as support for seasonally different migration strategies. However, the observed migration speed is the outcome of an underlying strategy, e.g. time- or energy-selected migration, and it could well be that a time-minimizing bird migrates slower than an energy minimizer if resources available in the environment are insufficient [32,38]. The overall migration speed was indeed higher in spring than in autumn, while the seasonal response patterns to different resource gradients showed that the birds adhered to a time-minimization strategy in both seasons. The longer overall duration (and slower overall speed) of migration in autumn compared with spring is partly or fully a combination of a relatively long mid-migration stop in autumn, when birds likely initiate moult [44]. Also, the longer initial flights in spring suggest that pre-flight fuelling times were longer in spring than in autumn, further contributing to an apparent higher migration speed in spring.

A few previous studies of stopover behaviour in shorebirds at one site found support for a time-minimizing strategy in spring [5,14,45]. Stopover durations and departure fuel loads from James Bay, Ontario, Canada, in autumn indicated that species with the longest migrations (greater than 9000 km) were consistent with a time-minimization strategy, while shorter-distance migrants (*ca* 5000 km) showed characteristics of energy minimization [46]. Considering the severe time constraints in long-distance migrants, where migration may constitute up to 50% of the non-breeding time budget, the incentive for minimization of the time for migration in both seasons seems logical. Not only do birds need to find time to moult and recover from physiological strains associated with migration during the non-breeding period, but a fast migration also minimizes the time exposed to elevated mortality risk during this period [47–49].

### (b) Flight strategy

The observed flight pattern in the LRP suggests that birds often depart on migratory flights sufficiently loaded to allow several flights in rapid succession without intermediate fuelling. The arrangement of migratory flights in blocks of back-to-back flights appears to occur also in landbirds [2,3], a pattern that should not be mixed up with overloading *sensu* Gudmundsson *et al.* [14]. It may seem counterproductive for a time-minimizing migrant to arrange migratory flights as runs of two to seven flights with short intermediate daytime stops. It is unlikely that significant refuelling takes place during these daytime lay-overs, but perhaps the birds can cover the field metabolic costs by feeding and thereby avoid nibbling into the fuel load. At any rate, they will lose some time stopping during daytime and so there could be some other reason(s) for avoiding long-haul flights, although non-stop flights of up to 48 h did occur (and 60 h for combined back-to-back runs of flights). Nocturnal migratory flights are beneficial in visual-guided foragers (like LRP) since potential foraging time in daylight is not compromised [50], although this assumes they can gain fuel effectively during daytime. There could be other factors, unrelated to foraging and fuelling, that make birds refrain from continuing flying in daytime after a nocturnal flight. These may include to avoid flying in turbulent conditions that prevail in daytime [51,52], avoid attacks from aerial predators focused on migratory birds [49,53], or avoid dehydration through elevated evaporative water loss during daytime in high ambient air temperatures [54,55]. Daytime flight may incur the risk of overheating due to sun irradiation, which has been suggested as a potential reason why great snipe, *Gallinago media*, flying non-stop between Scandinavia and Africa shift to high altitudes (6–8000 m) in daytime, where ambient temperatures are low [56]. Still another possible factor could be that the resource landscape is less homogeneous than assumed, where ‘soft barriers' of reduced resource availability could potentially generate the observed pattern [50]. The few greater than 24 hour flights that did occur could simply refer to opportunistic flight prolongation in favourable wind conditions [57].

A second characteristic of time-minimization migration is the adaptive adjustment of flight speed in relation to energy (fuel) deposition rate at stopovers [9]. Higher observed flight speeds in spring than in autumn have been taken as indication of seasonally different migration strategies, i.e. time minimization in spring and energy minimization in autumn [30,40,58]. We did not measure flight speed directly, but total autumn and spring flight durations were very similar. The fact that the estimated migration distance was about 6% longer in autumn than in spring suggests that, if anything, flight speed in spring was lower than in autumn. The mean flight speed estimated for the entire migration (14.7 m s^−1^) was remarkably close to direct measurements of flight speed in LRP (15.8 m s^−1^ (*N* = 3, s.d. = 0.75; unpublished ornithodolite measurements, 2013–2019; [59]).

Flight speed and hence duration of total flight time may be affected by external factors, such as flock size [60] and prevailing winds [61,62]. The spatio-temporal resolution of our data prevents a detailed analysis of potential wind support, and we therefore provisionally accept the hypothesis that flight speeds appear to be similar in autumn and spring, which is to be expected by birds adopting the same (time-minimizing) strategy [9].

## 5. Conclusion

Researchers have recently drawn attention to possible alternative ‘functions’ of stopovers in migratory birds [63,64]. There may be many reasons why birds decide to stop (or interrupt) a migratory flight other than running out of fuel, or to refrain from departing from a stopover even if the optimal fuel load has been reached. Such factors include adverse weather and winds [65–67], the need to sleep [68], involvement in social interactions, and recovery from physiological imbalance [63] and suppressed immune function [69]. In the case of the LRP we found that most birds made a relatively long autumn mid-migration break, which is also the case in LRPs breeding in Finland and migrating to southern India [70]. During this period, which on average exceeded seven weeks, the birds likely initiate their annual moult (cf. [44]). All such factors will contribute to a weakened association between fuelling rate, stopover duration and flight step length as predicted from OMT. Yet, we find strong relations between the fundamental parameters of OMT when analysing stopover–flight dynamics for a long-distance migratory shorebird. This emphasizes the prime function of stopovers as episodes of refuelling for subsequent flight(s). Our approach shows a novel way to use biologging data to evaluate which of alternative migration strategies a species (or population) adopt. We hope that similar efforts for other species and populations will follow to reveal if time minimization is universal among long-distance migrating birds and seasons or find cases where alternative strategies are used.

## Data Availability

The data on which this study is based are available from the Dryad Digital Repository [71]. Supplementary material is available online [72].
